# Impact of telemonitoring approaches on integrated HIV and TB diagnosis and treatment interventions in sub-Saharan Africa: a scoping review

**DOI:** 10.15171/hpp.2017.12

**Published:** 2017-03-05

**Authors:** Clarence S Yah, Ernest Tambo, Christopher Khayeka-Wandabwa, Jeanne Y. Ngogang

**Affiliations:** ^1^WITS Reproductive & HIV Institute, Faculty of Health Sciences, University of the Witwatersrand, Johannesburg, South Africa; ^2^Department of Biochemistry & Microbiology, Nelson Mandela Metropolitan University, South Africa; ^3^Department Biochemistry and Pharmaceutical Sciences, Higher Institute of Health Sciences, Université des Montagnes, Bangangté, Cameroon; ^4^Africa Disease Intelligence and Surveillance, Communication and Response (Africa DISCoR) Institute, Yaoundé, Cameroon; ^5^African Population and Health Research Center (APHRC), Nairobi, Kenya

**Keywords:** Telemonitoring, Empowerment, HIV/TB, Access, Uptake, Sustained, Interventions

## Abstract

**Background:** This paper explores telemonitoring/mhealth approaches as a promising real time and contextual strategy in overhauling HIV and TB interventions quality access and uptake, retention,adherence and coverage impact in endemic and prone-epidemic prevention and control in sub-Sahara Africa.

**Methods:** The scoping review method was applied in acknowledged journals indexing platforms including Medline, Embase, Global Health, PubMed, MeSH PsycInfo, Scopus and Google Scholar to identify relevant articles pertaining to telemonitoring as a proxy surrogate method in reinforcing sustainability of HIV/TB prevention/treatment interventions in sub-Saharan Africa. Full papers were assessed and those selected that fosters evidence on telemonitoring/mhealth diagnosis, treatment approaches and strategies in HIV and TB prevention and control were synthesized and analyzed.

**Results:** We found telemonitoring/mhealth approach as a more efficient and sustained proxy in HIV and TB risk reduction strategies for early diagnosis and prompt quality clinical outcomes. It can significantly contribute to decreasing health systems/patients cost, long waiting time in clinics, hospital visits, travels and time off/on from work. Improved integrated HIV and TB telemonitoring systems sustainability hold great promise in health systems strengthening including patient centered early diagnosis and care delivery systems, uptake and retention to medications/services and improving patients’ survival and quality of life.

**Conclusion:** Telemonitoring/mhealth (electronic phone text/video/materials messaging)acceptability, access and uptake are crucial in monitoring and improving uptake, retention,adherence and coverage in both local and national integrated HIV and TB programs and interventions. Moreover, telemonitoring is crucial in patient-providers-health professional partnership, real-time quality care and service delivery, antiretroviral and anti-tuberculous drugs improvement, susceptibility monitoring and prescription choice, reinforcing cost effective HIV and TB integrated therapy model and survival rate.

## Introduction

HIV and tuberculosis (TB) treatment and prevention programs, remain a challenge in endemic region due to insufficient electronic technology to support the uptake and compliance.^1^ Based on the reported evidence-based and theoretical information from developed countries, the positive behavioral impact on the use of social media and mobile messaging health (mhealth) has been shown as a form of pervasive health interventions and communications.^2^ In addition, over 80% of people globally make use of mobile or electronic health technology and applications for information communication (short texting messaging) which can be used to improve healthcare and service delivery in remote areas.^3^ However, the use of mhealth to store, analyze, and share patient information “for public health data triangulation remain a challenge in sub-Saharan Africa due to limited infrastructures, awareness and policies.^4^ Evidence based interventions have shown the use of mobile/electronic technology associated with increasing awareness of patient health education, adherence, counseling, dual patient-provider participation and feedback on medications and safety.^2,5^

Telemonitoring systems are used as proxy rationalized technology that may support, store, analyze, and share patient data/information needed in maximizing evidence-informed health decisions.^1,3-5^ These systems may also be used for service delivery strategies, point of care or home-based service delivery, good health performance and effective strengthening of healthcare systems and workforce.^2,5^ Despite these advantages, there is little or no evidence based documentation in sub Saharan Africa that expand knowledge of telemonitoring as surrogate expedite platform for health education, awareness, motivation and medication adherence among people living with HIV, TB, non-communicable diseases and other opportunistic infections^6^ in sub-Saharan Africa.

This has restricted the integration of telemonitoring strategies to enhance HIV and TB sustained interventions uptake, compliance and coverage to empower positive behavioural changes in most settingsa.^4-7^ Thus, resulting into insufficient data and information to support com­munity-based intervention programs, preventative and curative service deliveries across sub-Saharan Africa.^1,6,7^ Developing countries especially sub-Saharan Africa which is burdened with various emerging and persistent endemic diseases need evidence cutting-edge scientific and technological advancements in improving healthy lifestyle and wellbeing. Sub-Saharan Africa needs innovative technology applications and tools (e.g., mhealth or ehealth approach) that facilitate effective and efficient epidemiologic, clinical and behavioral surveillance data collection for evidence-based health systems thereby reengineering resilient behavioural improvements. Digital and social media innovations such as mobile phones electronic and internet chatting, televideo and messaging integrated into primary healthcare policies of safety and precautionary measures, awareness, consultations and counseling can ease open access and enhance patient care, treatment, adherence, monitoring as well as open access to patient-data sharing amongst professionals and providers thereby improving quality of health, life and productivity.^1,3-5^

Telemonitoring expatiated as the use of mhealth or ehealth approaches (mobile phone text/video/materials messaging/ social media platform connection/call) to access, monitor and improve uptake, retention adherence and coverage of HIV and TB programs effectiveness remain valuable expedite tool in health systems strengthening. This has been used in various settings to monitor and improve patient outcomes accounting in significant reductions in patients waiting time in hospital settings,^8^ whereas only few pilot studies have been conducted in selected Africa countries.^4,9^ The impact of telemonitoring is unequivocal in these settings.^8^ It may be helpful in expansion and scaling up healthcare services delivery, revamping community health workers and allied workforce performance and efficiency, reaching remote and hard to reach communities. Likewise, assists in increasing mutual dialogue and participation between patient and care provider and health professionals, thus, lessening the challenges of traditional healthcare face to face and onsite, as well as distant counseling and diagnosis, treatment of patients by health care providers.^2,6,8,10^ Telemonitoring has been proven as a cheap, potential and sustained health care delivery process when compared to traditional standards.^10^ For example, telemonitoring has been found to reduce hospital saving from €40 397.00 per patient to less than €36 802.97 among cystic fibrosis patients.^10^

This paper therefore explored the promising of real-time and contextual telemonitoring systems in revamping HIV, TB and national immunization access, retention, adherence and coverage programs in sub-Saharan Africa settings and other endemic-prone countries.

## Methods

To ascertain the importance of telemonitoring approaches in reinforcing sustainability of HIV/TB prevention/treatment interventions in sub-Saharan Africa, authors used various journal indexing platforms to access articles describing electronic and messaging health technology that enhances patient curative care, quality of care and healthcare policies relevant to African settings. The scoping review design-method was applied to synthesize the information extracted from indexed journal research hubs. Medline, Pubmed, MeSH PsycInfo, Scopus and Google Scholar journal indexing bodies were used to assess and identify the relevant telemonitoring articles. The search terms used in locating relevant articles were “Telemedicine community engagement HIV/TB, or telemonitoring community engagement HIV/TB or mhealth community engagement HIV/TB or electronic messaging community engagement HIV/TB” tactics in social behavioural changes. The reference articles were those published in English and French languages only.

## Results and Discussion

### Strengthening participatory community engagement telemonitoring HIV/TB prevention and retention program in sub-Saharan African

**Figure 1:**
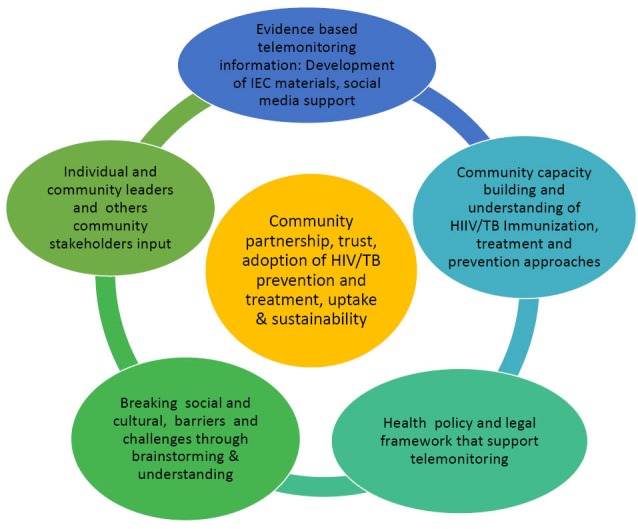
Participatory ecological framework of integrated HIV and TB telemonitoring intervention cascade. The invasion and infusion of telemonitoring/mhealth information technology into health care services has revamped and reduced health burden.

Understanding the local context, cultural norms and ecological issues and challenges can play a significant role in the design and integration of HIV and TB telemonitoring programs. The spectrum of cultural and socio-ecological engagement can lead to integrating HIV/TB prevention into the community life style.^11,12^ Building the enabling environment, community partnership and participatory community engagement (PCE) are essential for long-term access, trust and uptake of innovative HIV/TB telemedicine approaches and interventions as shown in Figure 1.

The prevention cascade can be achieved by targeting the entire cultural and socio-ecological framework cascade that reduces risk perception/awareness and enhances acceptability and adoption of the prevention/treatment approaches.^11,12^ The local socio-cultural ecological cascades in this context are trusted community delivery platforms that are policy-driven and ensure HIV and TB prevention and treatment interventions in reaching the target populations. These may include the access and uptake coverage to quality programs and strategies coupled with ownership, shared accountability and transparency.

There is an urgent need to increase collective community engagement in adoption, acceptability and sustained telemonitoring uptake approaches that initiate and expand early HIV and TB prevention and treatment programs. Community dialogue and PCE have been vindicated and proven as programs to support integrated HIV/AIDS and TB telemonitoring platforms.^13^ Hence, contextual evidence-based premises such as PCE are cost effective and essential entry points that support and addresses health interventions uptake at various levels of the ecological framework, compared to the existing conventional methods of care delivery.^11^ For example, local ecological cascade has been used in addressing programs among hard to reach men who have sex with men in resistant communities.^14^ Based on the PCE ecological model, community interventions have been found associated with greater awareness and healthier behavioral outcomes.^15^

Implementing telemonitoring approaches, framework and interventions of HIV and TB prevention holds great promises and opportunities in strengthening HIV and TB prevention and subsequently healthier communities’ lifestyles.^9,15^ Therefore, an effective uptake and treatment of TB and HIV or immunization program coverage is a function of innovative and sustained stakeholders’ engagement and leadership. The increase sensitization, awareness, participation and motivation of stakeholders in PCE^16^ play a significant role especially in designing quality-driven programs, recruitment and programs implementation. For example, the use of mobile phones and social media are common communication technological phenomenon in Africa today. More to that, in sub-Saharan Africa there is hardly no family without a mobile or social media facility^3^ which can be adapted and used in mobile health monitoring. Several mobile health technology platform including android/smartphones or iPads-enabled devices using Open Data Kit (ODK) software format such as video, audio, visual and text messaging may be helpful in modifying the disease surveillance and monitoring.^9^ To ensure the effectiveness, appropriateness and acceptability of the integrated HIV/TB telemonitoring, PCE must be properly planned with coordinated implementation.^3^ Therefore, strengthening community engagement uptake and retention toward ownership processes must be implemented with proper culturally dignified approach, local trust and accountability. The use of PCE can act as pathway to systematic evaluation of process indicators, outcomes and impact of HIV and TB treatment and prevention programs. For example, coverage and uptake of HIV and TB surveillance and monitoring depends on the sustained uptake and adoption of the intervention at various stages of the stakeholder engagement.^16^ This depends on the PCE fidelity, integrity, honesty, community respect/trust and the delivery of the program.

The engagement of communities with stigmatized diseases such as HIV and TB in health promotion programs is not an easy process. It requires appropriate and culturally improved communication skills as well as thought and buy-in planning. This is embedded in a constituted awareness and motivation meetings with diverse stakeholders including health officials, local community health workers, local and international non-profit organizations (NGOs), community leaders, faith-based organizations, stakeholders-policy makers, gate keepers, local women/ men association. This framework is based on trust and legal structures that enhances and maximizes programme strength, improvement as well as sustainability. Thus, such an engagement may increase public perception and altitude against fear and myth on HIV and TB co-infections using PCE strategy.^17^ In such PCE environment, the stigma and other structural factors including the questions on what the HIV and TB intervention is all about, and how it will be implemented and structured and the involvement of the community are discussed. Moreover, the functions and resources including benefits, time and dissemination of findings are also discussed in PCE.^14^ Implementing PCE can promote ethical and legal conduct on how interventions can be implemented successfully in local context including retention and adherence.^18^

### Mobile and media network telemonitoring in early HIV/TB diagnosis, immunization coverage and effectiveness in Africa

It is critical to note that vaccine hesitancy determinants (determinants in delay acceptance or refusal of vaccination despite availability of vaccination services) like education and socio-economic status do not influence hesitancy in only one direction,^18^ unlike the social determinants of health within which education is driven in one direction leading to better health outcomes. As previously demonstrated the individuals with higher level of education may be associated with both lower and higher levels of vaccine acceptance.^19,20^ The limiting factors that hinder immunization coverage across the globe and predominantly in Africa are majorly from hesitancy determinants matrix that encompass; (1) contextual influences- of historic, socio-cultural, environmental, health system/institutional, economic or political factors, (2) ecological influences arising from personal perception of the vaccine or social/peer environment and (3) fear and misconception related to vaccine and vaccination specific issues, such as polio immunization linked infertility issue in Northern Nigeria.^18^ Some other barriers may include vaccination schedule, mode of administration and reliability and/or the source of vaccine and/or vaccine related equipment drawbacks.^21^ Thus, HIV and TB teledata-sharing platform has the capacity to enhance the patient and providers awareness through constructive debates and taming out the patients and communities for hesitancy risk impact in attaining the global HIV/AIDS and End TB goals.^1^ With this background, fostering the benefit of integrated HIV and TB telemonitoring platform, policy and interventions is crucial to prevent new infections, increasing community resilience and programs ownership in early diagnosis and care seeking, packages uptake and quality life outcomes.^22-25^ Telemonitoring approaches implementation in integrated HIV and TB programs for provides real-time and immense opportunities in improving primary healthcare access and treatment adherence, as well. Improving treatment adherence may diminish the rising trend of new infections and multidrug resistance in urban and rural remote settings in sub-Saharan Africa.^25^

### Tackling rising new pattern and rate of HIV and TB infections in Africa

There is increasing concern on the rising new infections pattern and trend in the region which requires more proactive policy and action in scaling-up HIV and TB surveillance which may be helpful in access to preventive measures (like early screening and diagnosis, test usage and adherence to drug regimens across Africa).^1,3,6,18,19,25^ Furthermore, there is a growing body of evidence for raising the new HIV infections rate which may probably due to unsafe sexual intercourse and drug users, gender inequities in family and society disparities, cultural practices including child marriage, polygamy/concubine and condom user non satisfaction that exacerbate the spread of HIV and hindered health and socio-economic development. These contributions are poorly documented and unclear in several settings in Africa and require further operational investigations.^10,16,25^ In addition, strengthening quality access and care uptake interventions including modern contraceptive methods amongst female sex workers and tattooing, detainees, drugs abuse and men who inject non-sterile needles, male homosexuals is crucial. Boosting community engagement, awareness and empowerment on social, ecological and cultural-related aspects of exposure and vulnerability factors and promoters which are associated with persistent transmission dynamics is important in escalating the benefits of future health and economic programs.

Tackling the new trend and pattern of HIV and TB infections, drug and multiresistance emerging from social and behavioural, economic and cultural barriers and challenges to access, uptake and retention requires developing and scaling up new tactics capitalizing on effective early access to and use of mobile health and social media telemonitoring surveillance and control programs. Moreover, increasing the rate of pre-exposure prophylaxis (PrEP) and antiretroviral treatment (ART) intervention activities aiming at prevention or reduction of new HIV infection and opportunistic illnesses are in line with the global HIV agenda, 2030.^1,7,15^ Coordinated, appropriate and sustained efforts are needed to reduce drug use-related risk behaviors, including risky injection practices and unsafe sex, transactional sex, trading sex for drugs, sharing contaminated needles/blades in tattooing, homosexuality, and circumcision.^4,6,13,19,25^

Furthermore, promoting antimicrobial resistance stewardship in inappropriate use of antiretroviral and anti TB drugs as well as increasing adherence to treatment and preventive measures may help easy reaction against adverse ART. In addition, strengthening patients and their families in nosocomial TB preventive measures by health workers may be a good practice. The other best practices to prevent the HIV and TB-associated risks are increasing public awareness and free mental health telecounseling services, reducing the laboratory tests cost and the out of pocket medication as well as prohibiting stigmatization and discrimination of people living with HIV/AIDS and TB through social support systems.^16,17,20^ Timely access of comprehensive and coherent HIV/TB barriers and challenges data and information is core for improvement and guiding evidence-based policies, programs and services formulation and implementation. Likewise, improved integrated HIV/TB advocacy and care programs implementation that to support telemedicine and socio-behavioral programs and services such as early family planning, reproductive and sexual education and awareness, counseling pre and post HIV testing, HIV home-based and personalized care and/or follow-up care management, and psychosocial and legal services.

### Future priorities and directions opportunities 

(a) Investing real-time and contextual mobile health and social media HIV and TB open access surveillance and monitoring approaches in revamping sustainability of national TB immunization effectiveness and improving PrEP, as well as access to effective programs and interventions in endemic-prone settings.

(b) Promoting research and development (R&D) nvestment in telemonitoring technologies and applications innovations as key indicators for early monitoring of treatment failure and patient triage for appropriate and personalized intervention prioritization.

(c) Promoting national and regional integrated HIV and TB telemedicine and telemonitoring programs advocated and supported by African governments and local stakeholders.

(d) Strengthening telemonitoring capability ito improve the patients and care providers quality of life.

(e) Addressing telemonitoring implementation barriers and challenges that include patient privacy and confidentiality, data sharing, digital literacy and language barriers, unreliable connectivity and telecommunication coverage aimed accelerating patient and providers’ telemonitoring technologies, applications and approaches for diagnosis in clinical sectors.

## Conclusion

Telemonitoring and mhealth approaches are hold immerse future in real-time and promising array of opportunities in improving primary healthcare especially increasing access and long-term uptake of preventive and treatment including HIV/TB in Sub-Saharan Africa. Fostering telemonitoring science and practice adoption and implementation in chronic and emerging infectious diseases threat and epidemics including retention, adherence and sustained programs is imperative in increasing the quality and performance of service delivery in rural and urban settings. Therefore, investing in simple, cost-effective and reliable telemonitoring science and practice approach implementation is vital to promote and scale up acceptability, trust and performance in health systems strengthening, regulatory compliance, patient-health professional and care providers dialogue and feedback. Other areas include boosting social security such as medical insurance, billing and reimbursement scheme integrated into routine telemonitoring primary care and practices in sub Saharan Africa.

## Ethical Approval

Not applicable.

## Competing interests

The authors declare no conflict of interest.

## Authors’ contributions

ET conceived the idea and performed the initial literature and manuscript draft. ET, CSY and CKW supported with additional information. ET, CSY, CKW, GM and JYN provided further inputs. ET critically revised the manuscript. All authors read and approved the final manuscript.

## Disclaimer

The review does not reflect the official views or policies of any institution or body nor its planned design and construct but the views of the authors.

## Acknowledgments

We are grateful to the valuable discussions and inputs from our field staff and students.
